# Salivary telomere length in oral leukoplakia and oral squamous cell carcinoma: A preliminary case- control study

**DOI:** 10.1016/j.jobcr.2026.101522

**Published:** 2026-08-14

**Authors:** Anoushka Chauhan, Punnya V. Angadi, Bhushan B. Kulkarni, Mehreen S. Belawadi, Rashmi Patil, Karthiga Sakthi

**Affiliations:** a Department of Oral Pathology and Microbiology, KLE VK Institute of Dental Sciences and Hospital, KLE Academy of Higher Education and Research, Belagavi, Karnataka, India; b Dr. Prabhakar Kore Basic Science Research Centre (BSRC) and Jawaharlal Nehru Medical College, KLE Academy of Higher Education and Research, Belagavi, Karnataka, India; c Department of Oral and Maxillofacial Surgery, KLE VK Institute of Dental Sciences and Hospital, KLE Academy of Higher Education and Research, Belagavi, Karnataka, India; d Department of Public Health Dentistry, KLE VK Institute of Dental Sciences and Hospital, KLE Academy of Higher Education and Research, Belagavi, Karnataka, India

**Keywords:** Salivary biomarker, Oral potentially malignant disorder, Oral leukoplakia, Oral squamous cell carcinoma, Saliva, Relative Telomere length

## Abstract

**Purpose:**

To evaluate salivary telomere length as a potential non-invasive exploratory biomarker for early molecular alterations associated with oral leukoplakia and oral squamous cell carcinoma.

**Methods:**

Forty-five participants were initially recruited: 15 healthy controls, 15 leukoplakia cases, and 15 oral squamous cell carcinoma (OSCC) cases. Two milliliters of unstimulated saliva was collected, and genomic DNA was isolated using the QIAamp DNA Mini Kit (Qiagen, Hilden, Germany). Relative telomere length (RTL) was assessed using quantitative real-time PCR via the comparative $2^{-\Delta \Delta C_{t}}$ method. Given the non-normal distribution of RTL values (Shapiro–Wilk p < 0.001 in all groups), group variations were evaluated using the non-parametric Kruskal-Wallis test, with Bonferroni-corrected Mann–Whitney U tests for pairwise comparisons.

**Results:**

The mean relative telomere length (RTL) ratios $2^{-\Delta \Delta C_{t}}$ were 0.41 ± 1.00 for healthy controls, 0.48 ± 0.80 for leukoplakia, and 0.61 ± 0.87 for OSCC. The Kruskal-Wallis test confirmed no significant difference in RTL ranks across the cohorts (H(2) = 2.68, p = 0.261), and Bonferroni-corrected post hoc Mann–Whitney U comparisons showed no significant pairwise differences between any two groups (all p > 0.35), revealing a highly variable, non-significant upward trend in salivary relative telomere length from health to malignancy.

**Conclusion:**

Salivary telomere length exhibits substantial inter-individual variability and a non-significant increasing trend from healthy controls to oral leukoplakia and OSCC. These preliminary findings suggest that relative telomere length may not be a reliable standalone biomarker in its current form and warrants further validation in larger, longitudinal studies.

## 1 Introduction

Head and neck squamous cell carcinoma (HNSCC) comprises malignancies arising from the oral cavity, pharynx, larynx, nasal cavity, and salivary glands, and remains one of the most common cancers worldwide.^1^ Oral squamous cell carcinoma (OSCC) accounts for approximately 90% of oral malignancies and continues to represent a major public health burden, particularly in South Asian countries, where tobacco use, areca nut consumption, and alcohol are prevalent risk factors.^2^ Despite advances in surgery, radiotherapy, chemotherapy, and targeted therapies, the prognosis of OSCC remains poor because many patients present with advanced disease. Therefore, improving early detection and identifying reliable biomarkers remain major priorities in the field of oral cancer research.

Most OSCCs develop through a multistep process involving oral potentially malignant disorders (OPMDs). According to the latest consensus report from the WHO Collaborating Center for Oral Cancer, OPMDs comprise a heterogeneous group of clinical disorders associated with an increased risk of oral cancer development. These include leukoplakia, erythroplakia, proliferative verrucous leukoplakia, oral submucous fibrosis, oral lichen planus, oral lichenoid lesions, chronic graft-versus-host disease, and selected inherited disorders.^2^ Although only a proportion of these lesions progress to malignancy, early recognition and appropriate risk stratification remain fundamental for preventing progression to invasive carcinomas.

Among OPMDs, oral leukoplakia is the most frequently encountered lesion and carries a variable but clinically significant risk of malignant transformation. Histopathological assessment of oral epithelial dysplasia (OED) remains the gold standard for estimating this risk.^3^ However, dysplasia grading is limited by inter-observer variability, biological heterogeneity, and the inability to accurately predict malignant progression in every individual lesion. Consequently, there is a growing interest in identifying objective molecular biomarkers that can complement conventional histopathological evaluation and improve risk prediction.^3^^,^^4^

Recent studies have identified several molecular biomarkers with potential utility in predicting malignant transformation in oral potentially malignant disorders, further supporting the need for reliable biomarkers to improve risk stratification and early detection.^5^^,^^6^ Saliva has emerged as an attractive diagnostic biofluid because it can be collected non-invasively, repeatedly, and economically, while reflecting both local and systemic molecular alterations. Numerous salivary biomarkers, including proteins, messenger RNA, microRNA, DNA methylation markers, metabolites, extracellular vesicles, and inflammatory mediators, have been investigated for their role in the early detection of oral cancer.^7^ However, despite encouraging preliminary findings, no salivary biomarker has demonstrated sufficient diagnostic accuracy or reproducibility for routine clinical application, highlighting the need to identify reliable biomarkers for early disease detection and risk stratification.^8^^,^^9^

Telomeres are repetitive TTAGGG nucleotide sequences located at the ends of chromosomes that preserve chromosomal integrity and genomic stability during cell division.^10^^,^^11^ Progressive telomere shortening occurs with cellular aging and repeated replication, eventually leading to cellular senescence or apoptosis. Conversely, dysregulation of telomere maintenance via telomerase activation contributes to chromosomal instability, unlimited cellular proliferation, and carcinogenesis. Altered telomere length has been associated with several human malignancies, including oral cancer, suggesting that telomere biology may represent a useful indicator of early molecular alterations during carcinogenesis.^12^^,^^13^

Although telomere length has been extensively investigated in peripheral blood and tissue specimens, evidence regarding salivary telomere length in oral leukoplakia and OSCC remains limited. Considering the advantages of saliva as a liquid biopsy and the potential role of telomere shortening as an indicator of genomic instability, evaluating salivary telomere length may provide valuable insights into the early molecular alterations associated with oral carcinogenesis. Therefore, this preliminary case–control study aimed to evaluate salivary telomere length in healthy individuals, patients with oral leukoplakia, and patients with OSCC and investigate its potential as a non-invasive exploratory biomarker associated with early molecular changes during oral carcinogenesis.

## 2 Material & methods

### 2.1 Study design and participants

This observational case–control study initially enrolled 45 participants (Table 1) categorized into three groups: healthy controls (n = 15), leukoplakia (n = 15), and oral squamous cell carcinoma (OSCC) (n = 15). Following real-time PCR quality control auditing, two samples within the healthy control cohort failed to yield detectable target or reference gene signals and were excluded from the downstream analysis. The final dataset comprised 43 participants: 13 healthy controls, 15 with leukoplakia, and 15 with OSCC.

**Table 1:** Demographic details.

Characteristic	Healthy Controls (n = 15)	Leukoplakia (n = 15)	OSCC (n = 15)
Number of participants, n	15	15	15
Age range (years)	24–69	25–66	25–64
Male, n (%)	9 (60.0)	13 (86.7)	10 (66.7)
Female, n (%)	6 (40.0)	2 (13.3)	5 (33.3)
Predominant habit	None	Smoking and chewing	Tobacco chewing
Predominant sites	NA	Buccal mucosa	Buccal mucosa > Vestibule > Tongue

Two healthy control samples were excluded following qPCR quality control; therefore, statistical analyses were performed on 13 healthy controls.

Healthy controls had no oral lesions, history of OPMD, malignancy, or systemic disease. They were recruited consecutively from individuals attending the outpatient dental department for routine dental examinations or minor dental treatments. All controls underwent a comprehensive oral examination by an oral pathologist to confirm the absence of oral potentially malignant disorders, oral cancer, or any other oral mucosal lesions. Individuals with a history of OPMDs, malignancy, chronic systemic illness, or tobacco- or alcohol-related oral pathology were excluded. Participants with oral leukoplakia and oral squamous cell carcinoma were recruited based on clinical diagnoses recorded in the Department of Oral Medicine and Radiology. Individuals with systemic illnesses or prior cancer treatment were excluded. All patients included in these two groups had a tobacco-chewing habit. Histopathological confirmation of the study lesions was not performed as part of the clinical study protocol; therefore, clinicopathological correlations could not be assessed. Written informed consent was obtained from all participants prior to the sample collection.

The study was approved by the Institutional Ethics Committee (EC/NEW/INST/2021/2435- SL. NO. 287) and registered with the Clinical Trials Registry of India (CTRI/2024/08/072442 dated 13th August 2024).

### 2.2 Saliva collection

Unstimulated saliva (2 mL) was collected from participants who refrained from eating, drinking, or smoking for at least 30 min prior to collection. Samples were obtained by passive drooling into sterile containers and stored at −80 °C until further analysis.

### 2.3 DNA extraction

Genomic DNA was extracted using the QIAamp DNA Mini Kit (Qiagen, Hilden, Germany) following the manufacturer's instructions. The DNA concentration and purity were assessed using a NanoPhotometer N60 (Implen, Munich, Germany).

### 2.4 Telomere length measurement by qPCR

Relative telomere length was assessed using a quantitative polymerase chain reaction (qPCR) method as described by Joglekar et al. (2020),^14^ with minor modifications related to instrumentation and reagents, while maintaining the original assay design and comparative ΔΔCt approach.

qPCR was performed using a StepOne Plus Real-Time PCR System (Applied Biosystems, Foster City, CA, USA) with SYBR Green PCR Master Mix (Green® Premix Ex Taq II; Takara Bio Inc., Shiga, Japan). Each reaction had a total volume of 10 μL and included primers specific for telomeric repeats or the β-globin single-copy reference gene.

The primer sequences used were as follows:

• Telomere primers:
  o Tel A (5′-CGGTTTGTTTGGGTTTGGGTTTGGGTTTGGGTTTGGGTT-3′)
  o Tel B (5′-GGCTTGCCTTACCCTTACCCTTACCCTTACCCTTACCCT-3′)
• β-globin primers:
  o hbg1 (5′-GCTTCTGACACAACTGTGTTCACTAGC-3′)
  o hbg2 (5′-CACCAACTTCATCCACGTTCACC-3′)

The thermal cycling conditions included initial denaturation at 95 °C for 10 min, followed by 40 cycles of denaturation at 95 °C for 15 s and annealing/extension at 60 °C for 1 min. Amplification specificity was confirmed using a melt curve analysis. No-template controls (NTC) were included for both primer sets. Although fluorescent amplification curves were detected in the Telomere NTC wells due to clean primer-dimer formations characteristic of self-complementary telomeric repeat primers (Tm≈79°C), all reference gene NTC wells remained completely clean and undetermined. All clinical samples were processed on a single 96-well plate to minimize inter-run variations.

The qPCR workflow adhered to key Minimum Information for Publication of Quantitative Real-Time PCR Experiments (MIQE) guidelines,^15^ including melt curve validation and use of controls; however, technical replicates and amplification efficiency analysis were not performed.

### 2.5 Data analysis

$C_{t}$ values for telomere and β-globin reactions were obtained using StepOne software v2.3 with automatic baseline and threshold settings. Relative telomere length (RTL) was calculated using the comparative $2^{-\Delta \Delta C_{t}}$ method as described by Joglekar et al. (2020).^14^ $\Delta \Delta C_{t}$ was defined as the difference between the target telomere $C_{t}$ value and the single-copy reference β-globin $C_{t}$ value. To stabilize calculations against outliers, the baseline calibrator was set as the mathematical mean $\Delta C_{t}$ of the healthy control cohort. Final values are expressed as the non-linear $2^{-\Delta \Delta C_{t}}$ relative ratio, where higher numbers directly denote longer relative telomeric repeats.

Statistical analysis was performed using Python (SciPy v1.x and Pingouin). Relative telomere length (RTL) values were calculated for each sample using the standard non-linear formula $2^{-\Delta \Delta C_{t}}$. Descriptive statistics were expressed as median and interquartile range (IQR) for RTL, given its non-normal distribution, with the mean and standard deviation (SD) reported for reference. Two samples in the healthy control group had missing ΔΔCt values and were excluded listwise rather than being imputed. The normality of the RTL distribution within each group was assessed using the Shapiro-Wilk test, which was significant (p < 0.05) in all three groups, indicating a violation of the normality assumption required for one-way ANOVA. Homogeneity of variance was confirmed using Levene's test (p = 0.850). Given the non-normal distribution, differences in RTL among the three cohorts were analyzed using the non-parametric Kruskal-Wallis H test. Pairwise group comparisons were performed using the Mann-Whitney *U* test with Bonferroni correction for multiple comparisons. A two-tailed p-value < 0.05 was considered statistically significant.

## 3 Result

**Fig. 1:**
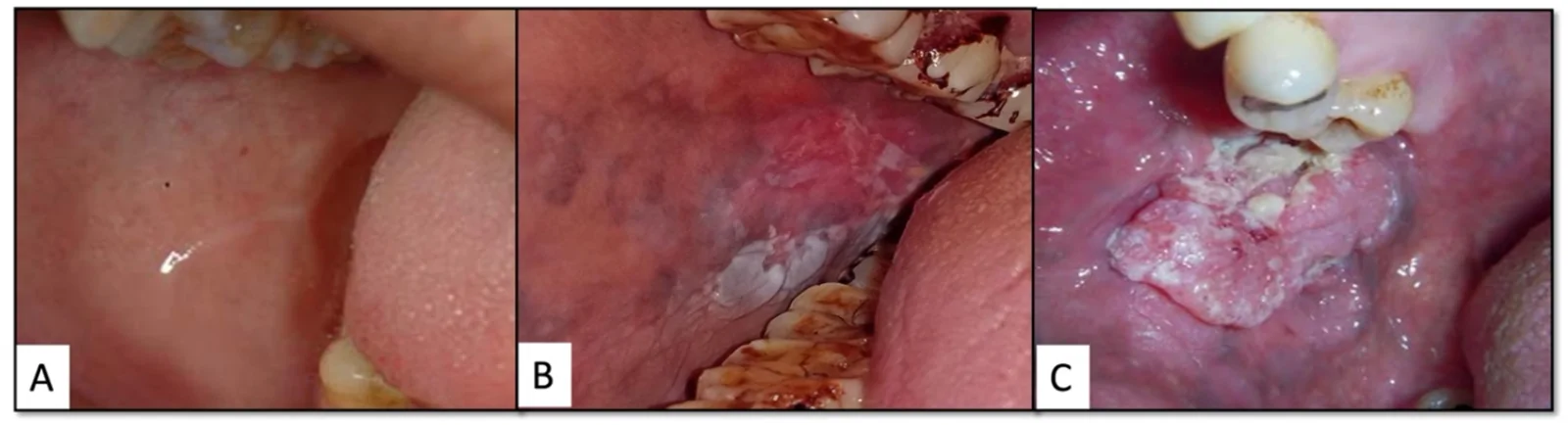
Clinical photographs of healthy controls (A), patients with oral leukoplakia (B), and oral squamous cell carcinoma (C).

A total of 45 participants were included in the study, comprising 15 healthy controls (9 males, 6 females; age range 24–69 years), 15 patients with leukoplakia (13 males, 2 females; age range 25–66 years), and 15 patients with oral squamous cell carcinoma (OSCC) (10 males, 5 females; age range 25–64 years) (Fig. 1) (Table 1). The lesions were located in the buccal mucosa, tongue, and gingiva. Among patients with leukoplakia, smoking was the most common habit, followed by tobacco chewing, whereas tobacco chewing was predominant among patients with OSCC, followed by gutka use. Following qPCR quality control, two healthy control samples were excluded because of undetectable target or reference gene amplification, resulting in a final analytical cohort of 43 participants (13 healthy controls, 15 with leukoplakia, and 15 with OSCC).

### 3.1 Salivary Telomere length analysis

**Fig. 2:**
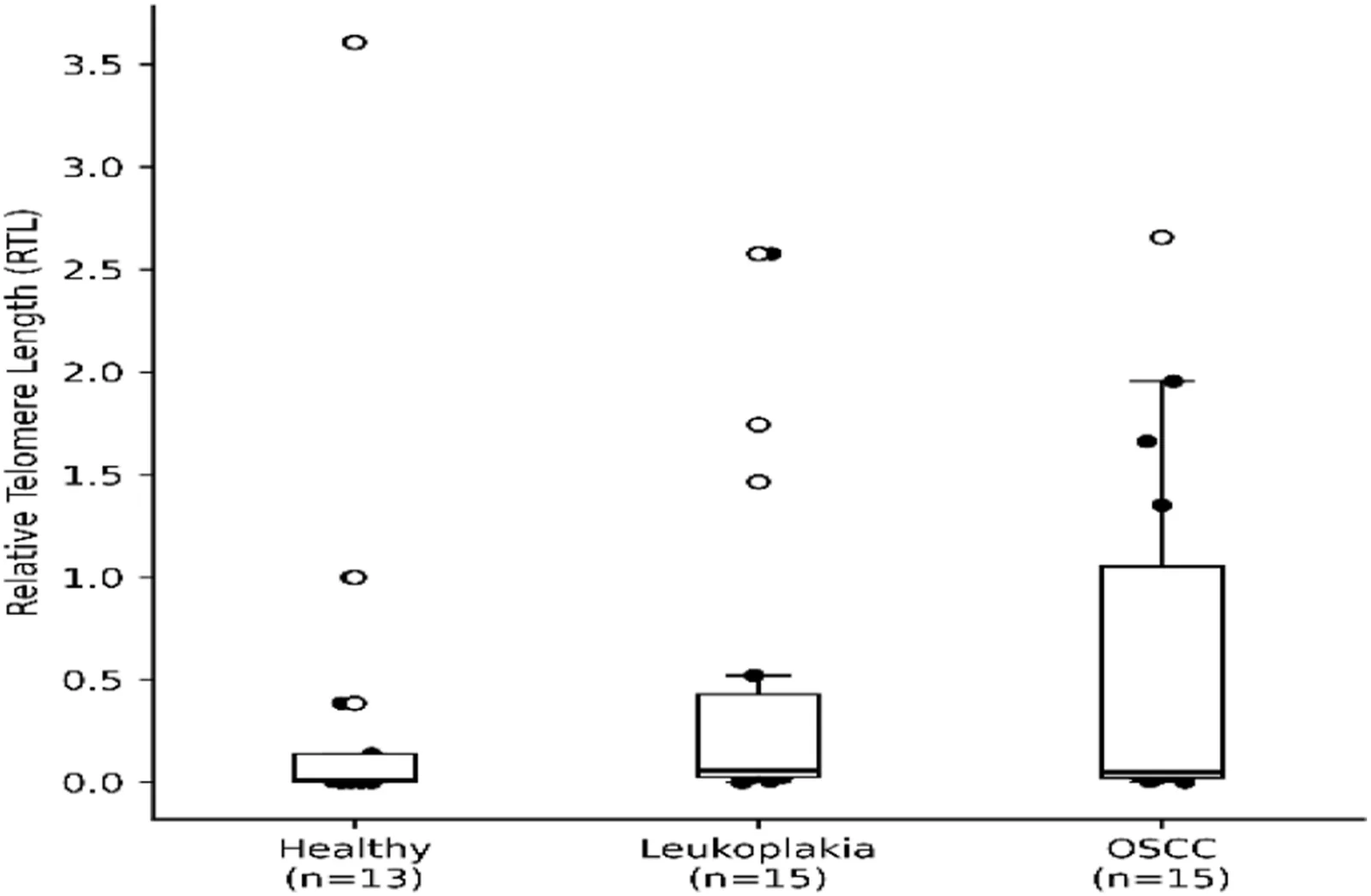
Distribution of salivary relative telomere length (RTL) among healthy controls (n = 13), oral leukoplakia (n = 15), and oral squamous cell carcinoma (OSCC) (n = 15). Box-and-whisker plots represent the median (central line), interquartile range (box), and whiskers representing the range of non-outlier observations, with individual participant values overlaid. Considerable overlap and inter-individual variability were observed across the three groups, consistent with the absence of a statistically significant difference in RTL (Kruskal–Wallis test, *p* = 0.261).

The individual raw $\Delta \Delta C_{t}$ values were converted to Relative Telomere Length (RTL) ratios using the formula $2^{-\Delta \Delta C_{t}}$. Because RTL values were markedly right-skewed and failed the Shapiro-Wilk test of normality in every group (p < 0.001), the median and IQR are reported as the primary summary statistics, with the mean ± SD given for reference. The median RTL value for the healthy control group (n = 13) was 0.0116 (IQR 0.0015–0.1393; mean ± SD 0.4055 ± 1.0020). For the oral leukoplakia cohort (n = 15), the median RTL was 0.0604 (IQR 0.0269–0.4296; mean ± SD,0.4751 ± 0.7967). The oral squamous cell carcinoma (OSCC) cohort (n = 15) exhibited a median RTL of 0.0516 (IQR 0.0230–1.0507; mean ± SD 0.6060 ± 0.8730) (Fig. 2).

Because RTL values were markedly right-skewed and failed the Shapiro-Wilk test of normality in every group (p < 0.001), the non-parametric Kruskal-Wallis test was used instead of one-way ANOVA (Table 2). This test showed no statistically significant difference in RTL among the three groups (H (2) = 2.68, p = 0.261). Post-hoc pairwise comparisons using the Mann-Whitney *U* test with Bonferroni correction likewise showed no statistically significant differences between any two groups (normal vs. leukoplakia, p = 0.748; leukoplakia vs. OSCC, p = 1.000; normal vs. OSCC, p = 0.352; Table 3).

**Table 2:** Descriptives and global cohort variations.

Diagnostic Group	Sample Size (n)	Mean RTL ($2^{-\Delta \Delta C_{t}}$​)	Median RTL (IQR)	Standard Deviation (SD)	Global Statistical Significance
**Healthy Controls**	13	0.4055	0.0116 (0.0015–0.1393)	1.0020	**Shapiro-Wilk:** p < 0.001 (non-normal); Levene's p = 0.850
**Oral Leukoplakia**	15	0.4751	0.0604 (0.0269–0.4296)	0.7967	**Kruskal-Wallis:** H(2) = 2.68, p = 0.261
**OSCC**	15	0.6060	0.0516 (0.0230–1.0507)	0.8730

**Table 3:** Pairwise post hoc comparisons (Mann–Whitney U, Bonferroni-corrected).

Comparison	Mann–Whitney U	p (uncorrected)	p (Bonferroni)	r value
**Normal vs Leukoplakia**	123.0	0.249	0.748 (NS)	−0.26
**Leukoplakia vs OSCC**	103.0	0.709	1.000 (NS)	0.08
**Normal vs OSCC**	63.0	0.117	0.352	0.35

NS: Not significant.

## 4 Discussion

Telomeres are repetitive DNA sequences located at the chromosomal termini that maintain genomic stability. Progressive telomere shortening during successive cell divisions normally triggers cellular senescence or apoptosis, whereas dysregulation of telomere maintenance through telomerase reactivation or the alternative lengthening of telomeres (ALT) pathway allows cells to bypass this checkpoint and contributes to genomic instability and tumorigenesis.^16^^,^^17^

The literature on telomere dynamics in oral carcinogenesis is notably not unanimous in its direction. A substantial body of work, including Sainger et al.,^18^ Aida et al.'s tissue and Q-FISH studies, and Zhu et al.'s population-level meta-analysis,^19^^,^^20^ reports that oral precancerous and cancerous tissues tend to show *shortened* telomeres relative to normal mucosa, consistent with the classical model in which attrition precedes and permits malignant transformation. In contrast, there is a separate line of evidence that is largely focused on telomerase activity and expression rather than length per se, reporting a progressive *increase* across the normal-to-precancer-to-cancer sequence. Samadi et al. found significantly higher telomerase activity in OSCC than in precancerous lesions and proposed that augmented telomerase expression, together with telomere length changes, contributes to OSCC progression and could serve as an early diagnostic biomarker.^21^ This is corroborated by immunohistochemical data showing that the hTERT labelling index and labelling score increase stepwise from normal oral mucosa through leukoplakia to OSCC,^22^ and by Rai et al.'s finding of elevated telomerase activity in OSCC relative to normal oral mucosal tissue.^23^ Pannone et al. similarly reported increased hTERT gene expression across the oral carcinogenesis continuum.^24^

In the present study, salivary telomere length was evaluated in patients with leukoplakia and OSCC relative to healthy controls. Contrary to our initial hypothesis of a linear reduction in telomere length during progressive transformation, our data revealed a highly variable, statistically non-significant upward trend in relative telomere length ratios (2^−^ΔΔCt) from healthy controls (0.41) to leukoplakia (0.48), and OSCC (0.61). Bonferroni-corrected post hoc pairwise comparisons (Mann–Whitney U) likewise showed no significant differences between any two groups (normal vs. leukoplakia, p = 0.748; leukoplakia vs. OSCC, p = 1.000; normal vs. OSCC, p = 0.352), reinforcing that the apparent upward trend does not reach significance at any stage of transformation. One possible explanation for this observation is the reactivation of telomerase or the engagement of alternative lengthening of telomeres (ALT) mechanisms during oral carcinogenesis. These mechanisms may contribute to the heterogeneous telomere length measurements in saliva. However, as telomerase activity and ALT were not evaluated in the present study, this explanation remains speculative and requires further investigation. Pal et al. observed a related phenomenon directly in oral leukoplakia, where telomeres in the lesion patch were somewhat longer and less consistent than those in paired normal oral mucosa, which they attributed to the localized reactivation of telomere maintenance mechanisms within the lesion.^25^ A similar mechanism may explain the variability observed in the present study.

The wide intra-group variation and higher mean RTL observed in the leukoplakia and OSCC groups may be explained by several factors. First, telomerase or ALT-pathway upregulation is a recognized hallmark of transformed and pre-transformed oral epithelial cells seeking to evade apoptosis, and can produce highly elongated or heterogeneous telomere distributions even within a single lesion.^18^ Second, whole saliva is a complex bioliquid containing not only exfoliated epithelial cells but also a substantial and variable influx of local inflammatory leukocytes and exudate, particularly under the chronic inflammatory conditions that accompany leukoplakia and OSCC. Because immune cells maintain comparatively long telomeres, their variable presence in mixed salivary DNA can mask epithelial-specific shortening and generate the kind of noisy, upward-skewed signal we observed.^26^

Additional contributors to telomere alteration likely include oxidative stress, chronic inflammation, and increased cellular turnover,^26^ with tobacco and betel quid use, the predominant habits in our leukoplakia and OSCC cohorts, further amplifying this oxidative damage.^27^ The utility of saliva as a diagnostic medium is supported by evidence that salivary telomere length correlates with peripheral blood telomere dynamics,^28^ reinforcing its plausibility as a non-invasive proxy for systemic and local telomere biology, even when the present whole-saliva measurements proved too heterogeneous to reach significance.

This study demonstrated a non-significant upward trend in relative salivary telomere length in oral leukoplakia and OSCC. Although this pattern is consistent with previous studies reporting increased telomerase activity during oral carcinogenesis, the present study did not assess telomerase activity or hTERT expression levels. Therefore, no conclusions regarding the underlying biological mechanisms can be drawn from these studies.

**Limitations.** The present study has limitations, including the relatively small sample size, cross-sectional design, absence of technical qPCR replicates and amplification efficiency analysis, and the heterogeneous cellular composition of whole saliva, which may have influenced the relative telomere length measurements. In addition, potential confounding factors, such as tobacco habits, age, and sex, were not adjusted for in the multivariable analyses. The absence of technical replicates, necessitated by the limited genomic DNA yield from low-volume saliva collections, is a further limitation of this exploratory pilot study. This was partially mitigated by running all target and reference reactions on a single 96-well plate to eliminate inter-plate variation, together with strict melt curve validation of every well. Consequently, the observed relative telomere length measurements should be regarded as preliminary and require validation in future studies that incorporate technical replicates and larger independent cohorts. Larger longitudinal studies incorporating technical replicates and cellular fractionation of salivary DNA paired with direct telomerase activity or hTERT expression assays are needed.

Conclusion: This preliminary case–control study evaluated the potential of salivary relative telomere length as a non-invasive biomarker for oral carcinogenesis. Although a non-significant increasing trend was observed from healthy controls to oral leukoplakia and OSCC, the substantial inter-individual variability and lack of statistical significance suggest that whole saliva relative telomere length is unlikely to serve as a reliable standalone biomarker in its current form. Nevertheless, these findings provide preliminary insights into telomere biology in oral carcinogenesis and warrant further validation in larger longitudinal studies incorporating technical replicates, cellular fractionation, and complementary assessments of telomerase activity or hTERT expression.

## Patient consent

All Patients provided a written informed consent for saliva collection.

## Ethical approval

The study was approved by the Institutional Ethics Committee (EC/NEW/INST/2021/2435- SL. NO. 287) and registered with the Clinical Trials Registry of India (CTRI/2024/08/072442 dated 13th August 2024).

## Author contributions

Punnya V Angadi and Anoushka Chauhan conceptualized and designed as well as conducted the study.

Bhushan B Kulkarni and Mehreen S Belawadi performed the laboratory procedures and methodology validation.

Rashmi Patil assisted with patient recruitment and sample collection.

Karthiga Sakthi assisted with statistics.

Anoushka Chauhan and Punnya Angadi drafted the manuscript.

All authors reviewed and approved the final manuscript.

## Declaration of generative AI in scientific writing

During the preparation of this work, the authors used AI tools for language refinement. The authors reviewed and edited the content and take full responsibility for the manuscript.

## Funding

This work was supported by the KAHER Postgraduate Research Grant (KAHER/RD//24-25/D-20122404) provided by the KLE Academy of Higher Education and Research (KAHER), Belagavi, India.

## Declaration of competing interests

The authors declare that they have no known competing financial interests or personal relationships that could have appeared to influence the work reported in this paper.

## References

[1] Barsouk A., Aluru J.S., Rawla P., Saginala K., Barsouk A. (2023). Epidemiology, risk factors, and prevention of head and neck squamous cell carcinoma. Med Sci.

[2] Warnakulasuriya S., Kujan O., Aguirre-Urizar J.M. (2021). Oral potentially malignant disorders: a consensus report from an international seminar on nomenclature and classification, convened by the WHO collaborating centre for oral cancer. Oral Dis.

[3] Odell E., Kujan O., Warnakulasuriya S., Sloan P. (2021). Oral epithelial dysplasia: Recognition, grading and clinical significance. Oral Dis.

[4] Warnakulasuriya S. (2018). Clinical features and presentation of oral potentially malignant disorders. Oral Surg Oral Med Oral Pathol Oral Radiol.

[5] Cai X., Zhang J., Zhang H., Li T. (2023 May 13). Biomarkers of malignant transformation in oral leukoplakia: from bench to bedside. J Zhejiang Univ - Sci B.

[6] Kavitha L., Ranganathan K., Shyam S., Fathima J.H.S., Umesh W., Warnakulasuriya S. (2022). Immunohistochemical biomarkers in oral submucous fibrosis: a scoping review. J Oral Pathol Med.

[7] Foy J.P., Bertolus C., Ortiz-Cuaran S. (2021). The promising diagnostic value of saliva for oral squamous cell carcinoma: current evidence and future directions. Cancers (Basel).

[8] Maciejowski J., de Lange T. (2017). Telomeres in cancer: tumour suppression and genome instability. Nat Rev Mol Cell Biol.

[9] Bau D.T., Lippman S.M., Xu E. (2013). Short telomere lengths in peripheral blood leukocytes are associated with increased risk of oral premalignant lesion and oral squamous cell carcinoma. Cancer.

[10] Andreikos D., Kyrodimos E., Kotsinas A., Chrysovergis A., Papacharalampous G.X. (2024). Association between telomere length and head and neck cancer risk: a systematic review and meta-analysis. Int J Mol Sci.

[11] Romano A., Di Stasio D., Petruzzi M. (2021). Noninvasive imaging methods to improve the diagnosis of oral carcinoma and its precursors: state of the art non-invasiveel of a three-step diagnostic process. Cancers (Basel).

[12] Abati S., Bramati C., Bondi S., Lissoni A., Trimarchi M. (2020). Oral cancer and precancer: a narrative review on the relevance of early diagnosis. Int J Environ Res Publ Health.

[13] Shanti R.M., Tanaka T., Stanton D.C. (2020). Oral biopsy techniques. Dermatol Clin.

[14] Joglekar M.V., Satoor S.N., Wong W.K.M., Cheng F., Ma R.C.W., Hardikar A.A. (2020). An optimised step-by-step protocol for measuring relative telomere length. Methods Protoc.

[15] Bustin S.A., Benes V., Garson J.A. (2009). The MIQE guidelines: minimum information for publication of quantitative real-time PCR experiments. Clin Chem.

[16] Yakob M., Fuentes L., Wang M.B., Abemayor E., Wong D.T. (2014). Salivary biomarkers for detection of oral squamous cell carcinoma — current state and recent advances. Curr Oral Health Rep.

[17] Sudbo J., Reith A. (2003). Which putatively pre-malignant oral lesions become oral cancers? Clinical relevance of early targeting of high-risk individuals. J Oral Pathol Med.

[18] Sainger R.N., Telang S.D., Shukla S.N., Patel P.S. (2007). Clinical significance of telomere length and associated proteins in oral cancer. Biomark Insights.

[19] Aida J., Kobayashi T., Saku T. (2012). Short telomeres in an oral precancerous lesion: Q-FISH analysis of leukoplakia. J Oral Pathol Med.

[20] Zhu X., Han W., Xue W. (2016). The association between telomere length and cancer risk in population studies. Sci Rep.

[21] Samadi F.M., Suhail S., Sonam M., Ahmad M.K., Chandra S., Saleem M. (2019). Telomerase in saliva: an assistant marker for oral squamous cell carcinoma. J Oral Maxillofac Pathol.

[22] Kannan S, et al. Immunohistochemical detection of human telomerase reverse transcriptase in oral cancer and pre-cancer. J Oral Maxillofac Pathol. doi:.10.4103/0970-9290.84281. 10.4103/0970-9290.84281 21891916

[23] Rai A., Naikmasur V.G., Sattur A. (2016). Quantificatprecanceromerase activity in normal oral mucosal tissue and oral squamous cell carcinoma. Int J Med Paediatr Oncol.

[24] Pannone G., De Maria S., Zamparese R. (2007). Prognostic value of human telomerase reverse transcriptase gene expression in oral carcinogenesis. Int J Oncol.

[25] Pal J., Rajput Y., Shrivastava S. (2022). A standalone approach to utilize telomere length measurement as a surveillance tool in oral leukoplakia. Mol Oncol.

[26] Gavia-García G., Rosado-Pérez J., Arista-Ugalde T.L., Aguiñiga-Sánchez I., Santiago-Osorio E., Mendoza-Núñez V.M. (2021). Telomere length and oxidative stress and its relation with metabolic syndrome components in ageing. Biology (Basel).

[27] Jiang X., Wu J., Wang J., Huang R. (2019). Tobacco and oral squamous cell carcinoma: a review of carcinogenic pathways. Tob Induc Dis.

[28] McLester-Davis L.W.Y., Estrada P., Hastings W.J. (2023). Cross-tissue telomere length correlations in healthy humans: a review and meta-analysis. Ageing Res Rev.

